# Deep neural networks capture texture sensitivity in V2

**DOI:** 10.1167/jov.20.7.21

**Published:** 2020-07-21

**Authors:** Md Nasir Uddin Laskar, Luis Gonzalo Sanchez Giraldo, Odelia Schwartz

**Affiliations:** Department of Computer Science, University of Miami, FL, USA; Department of Electrical and Computer Engineering, University of Kentucky, Lexington, KY, USA

**Keywords:** deep learning, mid-level vision, deep neural network, texture perception, visual area V2, visual cortex

## Abstract

Deep convolutional neural networks (CNNs) trained on visual objects have shown intriguing ability to predict some response properties of visual cortical neurons. However, the factors (e.g., if the model is trained or not, receptive field size) and computations (e.g., convolution, rectification, pooling, normalization) that give rise to such ability, at what level, and the role of intermediate processing stages in explaining changes that develop across areas of the cortical hierarchy are poorly understood. We focused on the sensitivity to textures as a paradigmatic example, since recent neurophysiology experiments provide rich data pointing to texture sensitivity in secondary (but not primary) visual cortex (V2). We initially explored the CNN without any fitting to the neural data and found that the first two layers of the CNN showed qualitative correspondence to the first two cortical areas in terms of texture sensitivity. We therefore developed a quantitative approach to select a population of CNN model neurons that best fits the brain neural recordings. We found that the CNN could develop compatibility to secondary cortex in the second layer following rectification and that this was improved following pooling but only mildly influenced by the local normalization operation. Higher layers of the CNN could further, though modestly, improve the compatibility with the V2 data. The compatibility was reduced when incorporating random rather than learned weights. Our results show that the CNN class of model is effective for capturing changes that develop across early areas of cortex, and has the potential to help identify the computations that give rise to hierarchical processing in the brain (code is available in GitHub).

## Introduction

The tremendous progress in machine learning has shown that deep convolutional neural networks (CNNs) trained on image classification are remarkably good at object and scene recognition (Krizhevsky, Sutskever, & Hinton, 2012; LeCun, Bengio, & Hinton, 2015). Although CNNs (LeCun et al., 1989; LeCun, Bottou, Bengio, & Haffner, 1998; Krizhevsky et al., 2012; Zeiler & Fergus, 2014) are only crudely matched to the hierarchical structure of the brain, such models have been intriguingly able to predict some aspects of cortical visual processing (Khaligh-Razavi & Kriegeskorte, 2014; Yamins et al., 2014; Kriegeskorte, 2015; Yamins & DiCarlo, 2016; Cichy, Khosla, Pantazis, Torralba, & Oliva, 2016; Güçlü & van Gerven, 2015; Pospisil, Pasupathy, & Bair, 2016; Cadieu et al., 2014; Cadena et al., 2019; Kindel, Christensen, & Zylberberg, 2019). Here we focus on a question that has been less explored, namely, understanding how the visual representation changes hierarchically across layers of the artificial network in comparison to early cortical areas. By considering early cortical areas with presumably fewer transformational stages, we seek to get a better handle on some fundamental questions that are not well understood. Focusing on texture sensitivity and cortical area V2 as a paradigmatic example, we ask: When does texture sensitivity and compatibility to V2 data first emerge in the CNN? What computations in the CNN are important? What happens as one proceeds along higher layers of the CNN? What is the importance of supervised training versus the architecture itself? The CNN class of model under consideration includes stacked linear and nonlinear computations that are widely used in modeling neural systems, such as convolution, rectification, pooling of model neurons, and local (divisive) response normalization.

Texture is a statistically defined repetitive homogeneous structure. Textures are common types of visual inputs in nature. Apart from their representation in V2, there is a rich history of studying texture representation in higher visual areas such as V4 (Merigan, 2000; Hanazawa & Komatsu, 2001; Arcizet, Jouffrais, & Girard, 2008; Nandy, Sharpee, Reynolds, & Mitchell, 2013; Okazawa, Tajima, & Komatsu, 2015; Okazawa, Tajima, & Komatsu, 2016) and texture perception in psychophysics (Julesz, 1975; Tamura, Mori, & Yamawaki, 1978; Julesz, 1981; Bergen & Adelson, 1988; Malik & Perona, 1990; Wallis et al., 2017). Visualization of image features in the CNN reveals that the second layer but not the first layer has some texture selectivity (Krizhevsky et al., 2012; Zeiler & Fergus, 2014). However, there has been little work on this for understanding if and how texture representation in a population of such model neurons relates to cortical measurements across early visual areas.

**Figure 1.**
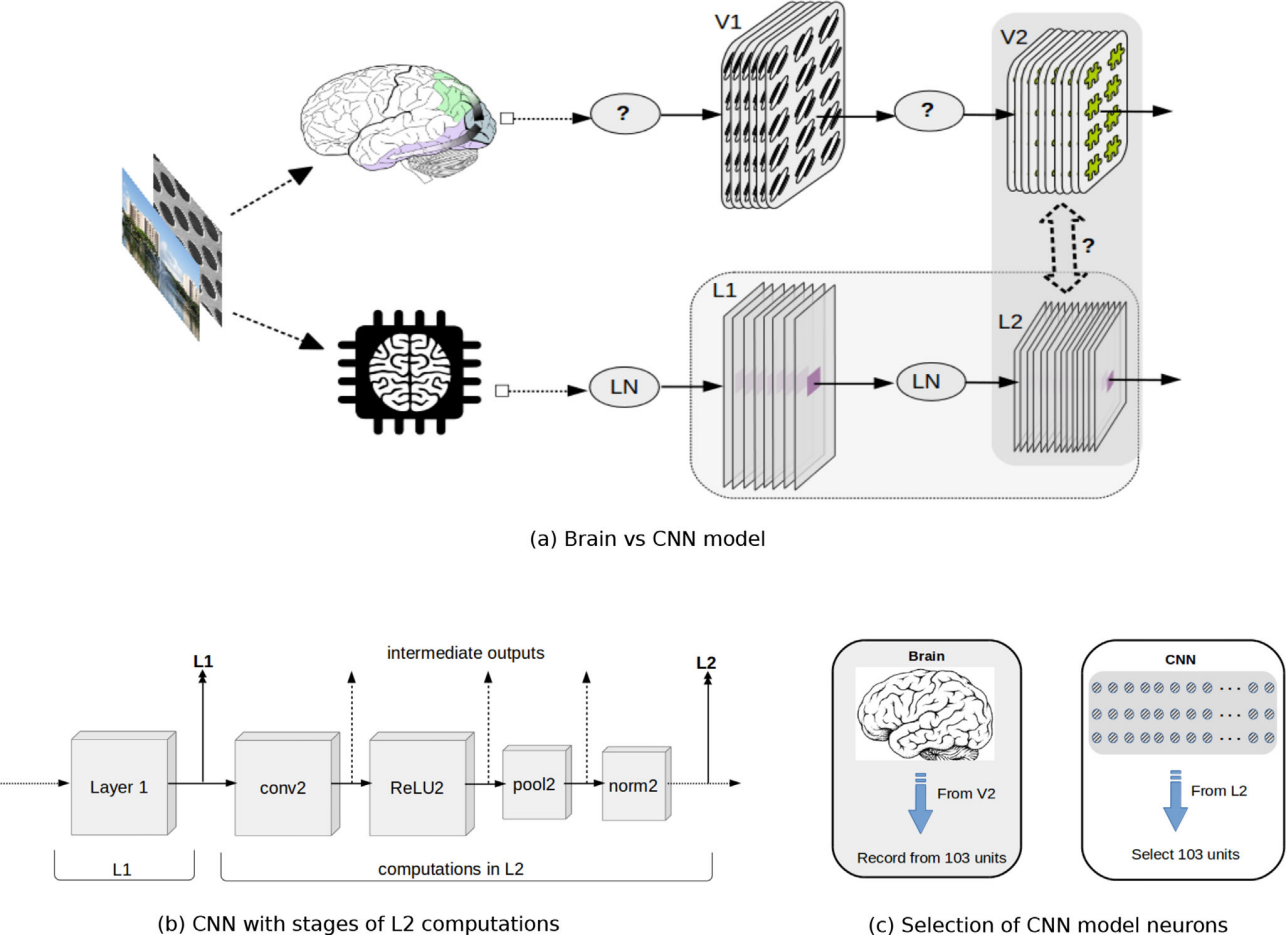
Simplified cartoon of hierarchical processing in the visual cortex and CNN. (a, top row): Cortical visual processing in the brain (only areas V1 and V2 are depicted). V1 has spatially oriented receptive fields (only one orientation is shown), but receptive fields in V2 are not yet clearly understood (hence puzzle symbol). The full linear nonlinear processing at each stage is not exactly known (hence the question mark) (Yamins & DiCarlo, 2016, Figure 1). (a, bottom row): Processing in CNNs. LN indicates linear and nonlinear transformations. The red box in the center represents the receptive field, the portion of the input visible to the model neuron. We ask whether there is correspondence between the representation in the CNN layers and in the cortical areas in the brain, especially between V2 and L2, although we also explore other layers of the CNN. (b) CNN with detailed intermediate linear and nonlinear computations in L2, from which we analyze the selectivity of the output at each stage. After convolution, the nonlinear transformations in the AlexNet (Krizhevsky et al., 2012) include a ReLU (Rectified Linear) nonlinearity, max pooling, and local response normalization loosely matched to divisive normalization models of V1 (Albrecht & Geisler, 1991; Geisler & Albrecht, 1992; Heeger, 1992). (c) Schematic of selection of 103 neurons from the brain recordings (e.g., from area V2). We select the same number of model neurons from the CNN (e.g., from layer L2) to find correspondence.

We focused on the transformation between primary visual cortex (V1) and secondary visual cortex (V2) as a baseline to compare the texture sensitivity at different layers of the CNN. The changes in representation between V1 and V2, and the computations that give rise to such changes, are not well understood. Recent experimental neurophysiology studies in macaque (and humans) have shown compelling analyses that cortical area V2, but not area V1, is sensitive to naturalistic textures (Freeman, Ziemba, Heeger, Simoncelli, & Movshon, 2013; Ziemba, Freeman, Movshon, & Simoncelli, 2016). Using both neurophysiology experiments and functional MRI, Freeman, Ziemba et al. (2013; Ziemba et al., 2016) have shown that high-order statistical dependencies of textures can differentiate V2 neurons from V1. We considered these data because they are currently the best test for distinguishing V1 from V2, and they provide a rich data set that could be compared to hierarchical models (Figure 1a).

In this article, we asked if hierarchical models such as the CNN can develop texture sensitivity and compatibility with the V2 texture data. We were particularly interested in what factors (if the model is trained or not, receptive field size of the model neurons, etc.) and computations (convolution, rectification, spatial pooling, normalization, etc.) (Figure 1b) are critical in doing so. We focused on the first two layers of the CNN, but additionally, we also considered the higher layers. This is because, the CNN architecture is only very loosely matched to cortex, and V2 responses may be better approximated with more layers.

We took AlexNet (Krizhevsky et al., 2012) as a popular example CNN model to show at what level a deep network starts to represent high-order statistics from textures, comparing to V2 in the brain. AlexNet has local normalization, a common computation used in neural modeling, unlike some other CNNs. As a point of comparison, we later considered another popular CNN used for cortical modeling, the VGG network (Simonyan & Zisserman, 2015), in which the receptive field size grows more slowly. In our approach, similar to the neurophysiology studies of Freeman et al. (2013), we were interested in pinpointing the layer at which texture sensitivity first emerged. Moreover, because we have more control over the different computational components in the CNN, we sought to also consider the particular computations within the layer. We concentrated on shallow (e.g., 4 to 8 layer) networks rather than very deep (e.g., even >100 layers) networks commonly used today in machine learning, since the cortical visual hierarchy itself is more shallow.

Without fitting to data, we found qualitative correspondence across a number of metrics between the first two layers of the CNN and the neuronal data. We also found some differences in the strength of effects between the CNN and the brain.

To fit the CNN model and data, we developed an approach for systematic quantification by selecting (Figure 1c) a population of CNN model neurons that best describes the primate brain recordings.

We were interested in both quantifying the compatibility and determining the main factors and computations that influence the compatibility. We found that the correspondence to V2 data first emerged at but not before the second layer of the CNN and specifically after rectification and improved after pooling. The compatibility was only mildly influenced by a nonlinear computation known as local response normalization (loosely matched to divisive normalization in V1) (Carandini & Heeger, 2012; Heeger, 1992; Albrecht & Geisler, 1991; Geisler & Albrecht, 1992). Higher layers in the CNN also could further improve the compatibility. The compatibility was reduced when incorporating random weights rather than the weights learned from images.

Our results show that the CNN class of model is effective for capturing changes across early areas of the cortical hierarchy. This more broadly presents the opportunity to go beyond demonstrating compatibility, to teasing out the computations that are important for hierarchical representation and processing in the brain. Our approach can be more widely applied to other related architectures, computational building blocks, stimuli, and neural areas.

## Introduction to the methods

### Computational building blocks of CNNs

In this section, we introduce the main methodologies for the convolutional neural networks, texture stimulus generation, and simulations. At the end of the article, we include a Technical Methods section. A number of hierarchical models in the machine learning and neuroscience literature include similar basic computational building blocks stacked together, namely, convolution, spatial pooling, rectification, and sometimes local response normalization. In this section, we describe the basic computational building blocks of convolutional neural networks.

#### Convolution

Deep convolutional neural networks include a linear front end, known as *convolution* in the neural network community. This corresponds to the cross-correlation between an input image and a filter.

Convolution has some properties that make it attractive to the deep learning community, namely, parameter sharing and the sparsity of connections. By sharing parameters across the whole input, convolution inherently learns location-invariant features. Convolution also ensures much less connections from the current layer to the next, which saves computational burden compared to typical fully connected artificial neural networks.

#### Max pooling

After convolution is performed on a layer input, a spatial pooling operation can be applied to the output map. The pooling in CNNs, including the ones we implement here, typically includes a max pooling operation, which only picks the maximum value within a pooling window. The size of the pooling window is a design choice. It is chosen based on the input shape, type, total number of layers in the network, and so on. The most common pooling window size is 2 × 2 or 3 × 3, which means that every max pooling operation will take the maximum value out of this window. The pooling window size can be different in different layers even in the same network.

Since the pooling window is applied to the image with shifts that are greater than one spatial unit (pixel, for instance), pooling shrinks the output maps progressively. In this sense, we can apply a larger number of filters as we move up in the hierarchy, keeping the number of parameters computationally manageable. At the same time, downsampling the feature maps has the effect of detecting a feature irrespective of its exact location. That is why pooling allows the CNN models to learn translation invariance and control overfitting.

#### Nonlinearity/activation functions

A point nonlinearity is applied to the output maps of max pooling. This allows the deep network models to learn robust nonlinear features from the previous layers instead of just their linear combinations. Modern CNNs typically include a rectified linear unit (ReLU) nonlinearity. These are also called the activation functions.

#### Local normalization

Local response normalization plays an important role in hierarchical object recognition models. Local response normalization is used in both Layer 1 and Layer 2 of AlexNet and has been shown to improve the recognition accuracy. It is loosely related to divisive normalization in the brain, by divisively normalizing the rectified response of a given neuron by the rectified responses of spatially overlapping receptive fields (five in the case of AlexNet). The CNN normalization differs from models of divisive normalization in neuroscience in two main ways: (a) Division by other neurons in the CNN is typically equally weighted, whereas models in visual neuroscience often include a weighted normalization signal (see the Discussion section), and (b) divisive normalization in the CNN typically includes only spatially overlapping filters, whereas models of normalization in visual neuroscience sometimes incorporate normalization from spatially surrounding filters to address surround modulation from beyond the classical receptive field (see the Discussion section).

If $a_{x,y}^{i}$ is the rectified linear activation at the (*x*, *y*) position in each *i*th channel, then the normalized response $b_{x,y}^{i}$ is defined by Krizhevsky et al. (2012):

(1) $$b_{x,y}^{i}=\frac{a_{x,y}^{i}}{{\left(k+\alpha \sum_{j=max(0,i-m/2)}^{min(N-1,i+m/2)}{\left(a_{x,y}^{j}\right)}^{2}\right)}^{\beta }}\;$$

where *m* is the size of the normalization neighborhood, and *N* is the total number of model units in the layer. Constants *k*, *m*, α, β are the hyperparameters with the default values of 2, 5, 10^−4^, and 0.75, respectively.

Normalization is done across the spatially overlapping unit activations across model units. Each 1 × 1 response is selected and normalized with corresponding values of all the model units across the channel dimension.

From a machine learning perspective, local response normalization is useful to normalize the unbounded activations coming from the ReLU (Rectified Linear) nonlinearity. It penalizes the responses that are uniformly large in a local neighborhood. It is a type of regularization that encourages competition among units in the network. Divisive normalization has been extensively studied in models of the visual system in neuroscience (Carandini & Heeger, 2012; Heeger, 1992; Albrecht & Geisler, 1991; Geisler & Albrecht, 1992).

### CNN training and simulations

The main simulations we ran followed the neurophysiology experiments with texture images in Freeman et al. (2013) and Ziemba et al. (2016) (simulation code is available in GitHub). We used CaffeNet, a variant of AlexNet (Krizhevsky et al., 2012), a popular deep CNN model widely applied in computer vision and neuroscience. Here we refer to the network as AlexNet. We chose AlexNet as our base model, because it includes computations that are loosely matched to visual cortex, such as pooling and local (divisive) response normalization. In addition, the receptive field (RF) size ratio can be controlled roughly to match the V1 to V2 ratio.

The CNN model was trained on the ILSVRC2012 ImageNet data set, a popular large-scale image database (Russakovsky et al., 2015). It is therefore important to note that the model was trained on natural images and then tested on texture images. This is crudely similar to what we envision for the brain. That is, the brain is exposed to natural images, but these images contain textures, and so the brain presumably develops texture sensitivity. Neurophysiology experiments have effectively probed sensitivity to textures along the cortical hierarchy, and here we probe the sensitivity in neural networks.

We took the layer outputs after pooling and normalization, referring to them as L1, L2, and so forth. To get a better handle on where exactly in the neural network compatibility with V2 first emerges, we also considered layer outputs at all other intermediate points of L2 in the network (Figure 1b). This allowed us to better understand how the computational building blocks (e.g., convolution, rectification, pooling, normalization) in the CNN may give rise to the differences observed in texture sensitivity between V1 and V2; in other words, at what point in the CNN there is a transition from V1-like behavior to V2-like behavior.

### Texture generation and neurophysiology data

The neurophysiology data for V1 and V2 are described in Freeman et al. (2013) & Ziemba et al. (2016), with recordings from macaque monkeys. We used the synthetic textures of Freeman et al. (2013), which were generated from a set of 15 real texture images. Each synthetic texture image was generated using the approach of Portilla & Simoncelli (2000). We refer to each set of textures generated from the same source image as *family* and all the images within the same family as *samples*. Naturalistic textures for a given family were generated each with a different random seed. Spectrally matched noise images (which we denote noise images) were generated by randomizing the phase of the synthetic images. The noise images have the same spatial frequency distribution of energy as the original ones but lack the differences in higher-order statistics.

Overall, the image set included 15 samples from each family, resulting in 225 texture and 225 noise images. We downsampled the textures so that the effective portion of the image that the CNN neurons are sensitive to is equated to the receptive field size of the cortical neurons. For more detail on this process and the texture generation, see Technical Methods.

We initially made a qualitative comparison between the cortical data and the CNN model. We then developed a quantitative approach to select a population of CNN subunits that were most compatible with the neurophsyiology data.

## Simulation results

### Qualitative correspondence of the CNN to the neurophysiology cortical data

We compared the cortical data and the CNN without any fitting, focusing on both visualization of the texture class clusters and a metric for texture sensitivity.

To make the CNN model and the cortical data more comparable, we equated the number of CNN model neurons in our simulations to the number of neurons in the neural population as in Freeman et al. (2013) & Ziemba et al. (2016) (102 V1 and 103 V2 neurons). We randomly selected 103 model neurons as shown in Figure 1c.

For the CNN, we considered the total number of filters in a given layer times a center 2 × 2 spatial neighborhood (see Technical Methods). We defined the spatial neighborhood as a population of neurons picked from the center of a layer output in the CNN. This is essentially a collection of neurons with spatial positions aligned with the center location of the input image. We considered a small spatial neighborhood so that the receptive fields of the model neurons lie in the main input but allow spatial jitter.

#### Visualization of texture clustering in the CNN population

To first gain intuition that L1 and L2 differ in their texture representation, we visualized the CNN model neuron population activity. We transformed the responses of CNN layers from a high-dimensional space (where dimensionality is the number of neurons in the given CNN layer) to a two-dimensional space. We used the Barnes-Hut *t*-distributed stochastic neighbor embedding (*t*-SNE) (van der Maaten, 2014; van der Maaten & Hinton, 2008) algorithm to achieve this visualization. *t*-SNE is a technique for dimensionality reduction that tries to model small pairwise distances to capture local data structures in a low-dimensional space.

**Figure 2.**
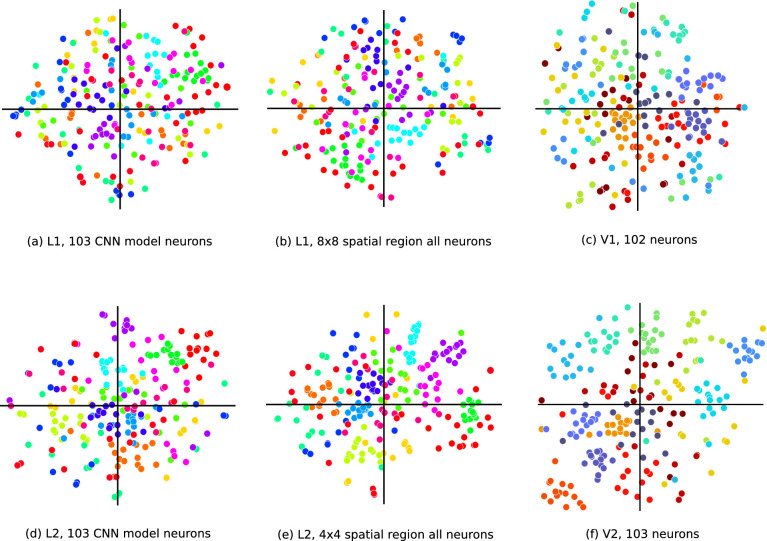
Qualitative depiction of texture class clustering in the CNN versus the cortical data. *t*-SNE visualization of CNN responses to natural textures. Each point represents a sample and each color represents a family. (a, d): The CNN L2 neurons are able to better separate the texture families than L1. This indicates that L2 neurons are more selective to the high-order texture properties of different families. (b, e): L2 neurons show superior clustering of textures even with fewer neurons than L1. (c, f): Cortical V1 and V2 neurophysiology data from Figure 4 in Ziemba et al. (2016). The CNN results are comparable to the neurophysiology data, but requiring in the CNN more than 103 neurons to obtain similar separation levels to the recorded V2 population.

In Figures 2A–E, each point results from embedding an image represented by a high-dimensional response vector into two dimensions. Therefore, we have a total of 225 points that come from the same number of images from 15 texture families. Each point represents the population response to a texture sample, and samples belonging to a same family share the same color. L1 responses are more scattered and do not group images of the same family tightly together. This is apparent both when randomly choosing 103 model neurons (Figure 2a) and when considering all neurons in an 8 × 8 spatial neighborhood (Figure 2b), amounting to a total of 3,072 neurons. In the L2 response space, samples from the same texture family group more tightly together. This is less clear when using 103 random neurons in L2 (Figure 2d) but becomes apparent when considering all neurons in a 4 × 4 spatial neighborhood (Figure 2e), amounting to a total of 2,048 neurons. L2 therefore better separates the texture responses than L1, qualitatively similar to what has been shown for V2 versus V1 in the neural data (Ziemba et al., 2016). However, a larger number of neurons from the CNN are required to match the texture discrimination capabilities of the V2 population.

#### Differentiating L2 neurons from L1 in CNNs via the modulation index metric

To further show the distinction between L1 and L2 for texture sensitivity, we followed the approach in Freeman et al. (2013) of computing a modulation index metric. The modulation index captures the differential response between textures and noise. We computed the mean modulation index for each of the 15 texture families, resulting in 15 mean modulation index values. This was done for each of the network layers, L1 and L2. We computed the modulation index from the responses of all samples from each family, both natural and noise, and averaged over the number of model neurons in the respective layer.

**Figure 3.**
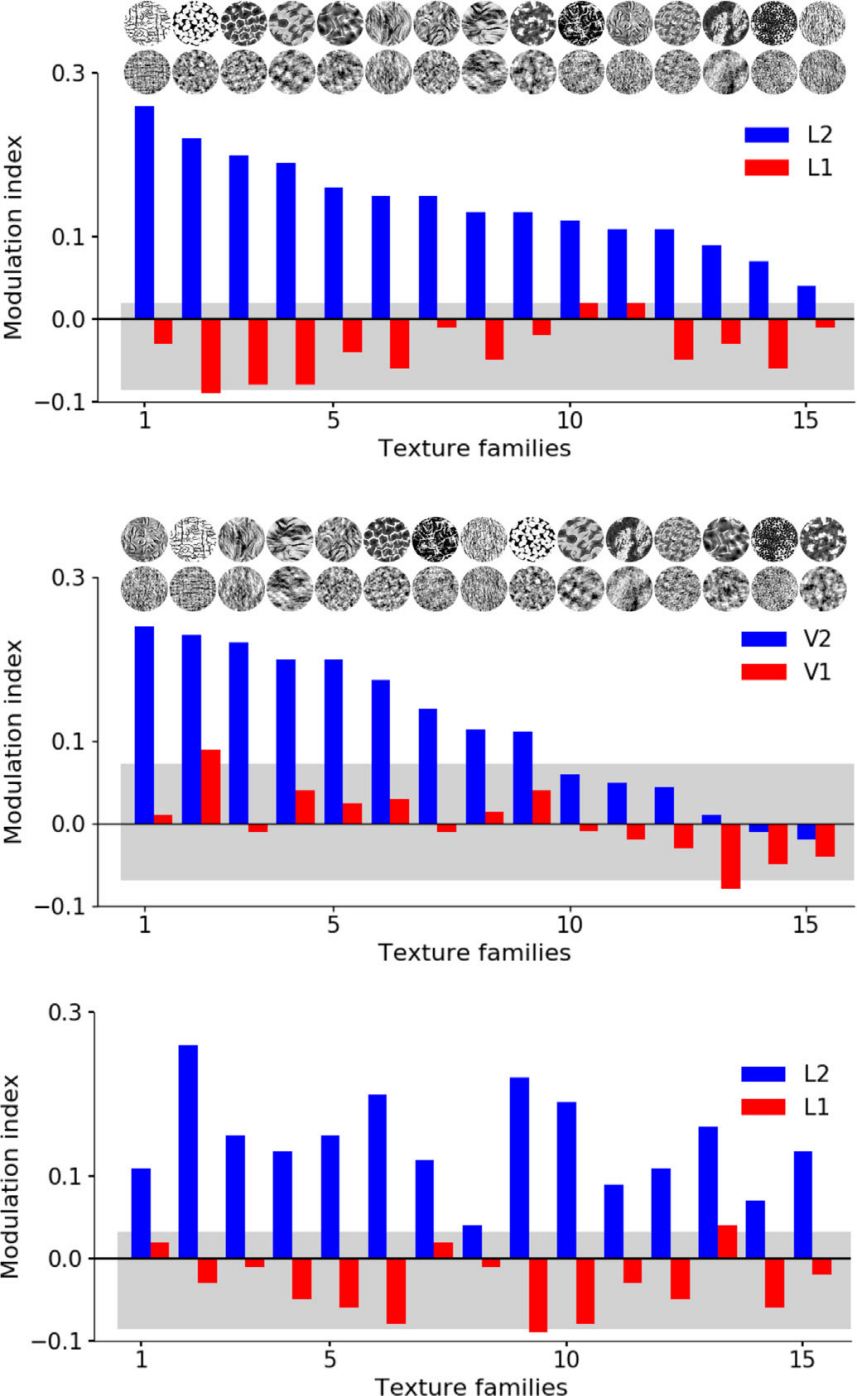
Modulation index in the CNN versus the cortical data. The modulation index is shown for each texture family, averaged over model neurons. Top panel: Data from the CNN, with the texture families sorted from high to low mean L2 modulation index. L2 (blue) neurons have higher modulation index than L1 (red) and hence higher differential response to the textures versus the noise. Light gray area shows the expected modulation due to chance and 2.5th and 97.5th percentiles of the null distribution of modulation. Corresponding textures and spectrally matched noise of the modulations are shown at the top. Middle panel: Data from the neurophysiology experiments of V2 and V1 (Figure 2e in Freeman et al., 2013), with the texture families sorted from high to low mean V2 modulation index. Bottom panel: Same CNN mean modulation indices as in the top panel, but with the texture families sorted according to the V2 mean modulation index data of the middle panel.

The modulation index M for each model neuron is defined as the difference of responses to the textures versus the noise, divided by their sum, according to (2):

(2) $$\begin{array}{c} M=\frac{r_{na}-r_{no}}{r_{na}+r_{no}},\; \end{array}$$

where *r*_*na*_ and *r*_*no*_ are the responses to naturalistic textures and noise, respectively. Figure 3 (top panel) shows the average modulation index for all texture families in the CNN, for L1 (*red*) and L2 (*blue*).

Averages are obtained from 10,000 repeats, where at each iteration, we randomly select 103 model neurons and compute the modulation index.

High modulation index of a population of model neurons toward a family means that this group of neurons is highly sensitive to this specific family; hence, they show high differential response. Since first- and second-order statistics are matched for both natural and noise images, a differential response also means that neurons latch onto higher-order statistical properties of the stimuli.

We found that the average modulation index of L1 model neurons is near zero and the modulation index of L2 is substantially higher than L1. The diversity in modulation index for the different texture families is shown in Figure 3, for both the CNN (Figure 3, top panel) and the neuronal data (Figure 3, middle panel). The average modulation index of L2 (0.18) is higher than L1 (−0.04). The difference between the indexes of L1 and L2 is significant (*p* < 0.0000005, *t* test considering signs; *p* < 0.00001, *t* test ignoring signs and considering only the magnitudes) and is qualitatively comparable but stronger than the neurophysiology data (V1: ≈0.00 and V2: ≈0.12; Freeman et al., 2013). More specifically, Figure 3 (top panel) shows that the L2 modulation is more drastic in some texture families than others, as also observed for the V2 data (Freeman et al., 2013). However, the rank order of the textures was different between the CNN and the neurophysiology data, which are shown in Figure 3 (bottom panel) (prompting our quantitative subset selection approaches below).

**Figure 4.**
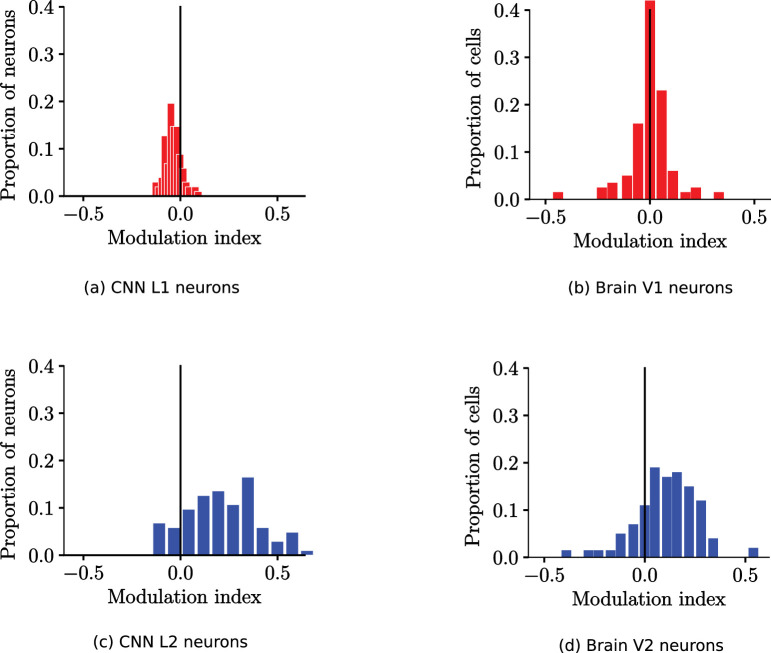
Distributions of modulation indices of model neurons versus the cortical data. (a) The CNN L1 modulation index centers toward zero, indicating that the model neurons respond with similar strength to the textures as to the noise. (c) A significant number of L2 model neurons have positive modulation indices, indicating that they respond more strongly to the textures than the noise. This trend of both L1 and L2 sensitivity is qualitatively compatible with V1 and V2, (b) and (d), respectively, in the brain (replotted from Figure 2(b) and (d), of Freeman et al., 2013).

Figure 4 shows the distribution of modulation index for each of the neurons in the CNN. Very few of the L1 model neurons (11%) have a positive modulation index (Figure 4a). In contrast, a significant portion of the L2 model neurons (94%) has a positive modulation index. This is an indication of L2 sensitivity toward the “naturalness” of textures (Figure 4b). This sensitivity is qualitatively similar to the neurophysiology results shown in Figure 2f of Freeman et al. (2013), which are reproduced in Figures 4c and 4d.

#### Modulation index for CNN with trained versus random weights

**Figure 5.**
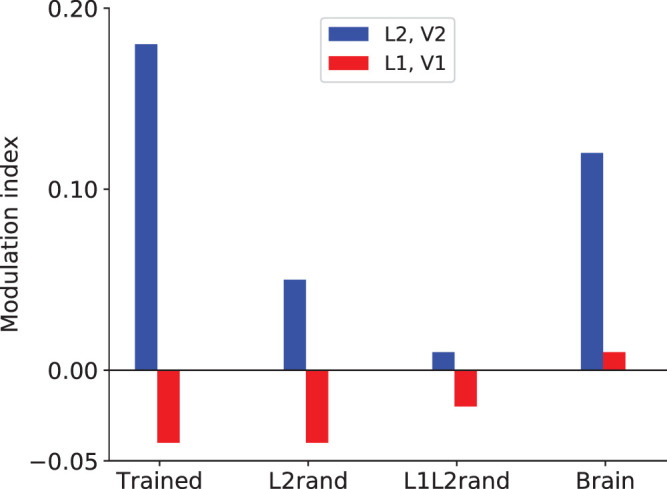
Effect of CNN model training on sensitivity to high-order texture properties in comparison to the brain data. Average modulation index for AlexNet model neurons with and without trained weights indicates the network with trained weights shows superior texture sensitivity according to the modulation index metric. Random weights in L2 (L2rand) significantly reduce the sensitivity of L2 neurons. In the complete absence of training in both layers (L1L2rand), texture sensitivity reduces even further.

We ran a control to examine the influence of the CNN architecture alone. Instead of using the model weights that resulted from training on the ImageNet database, we generated random weights (in the interval [−1, 1]) for the L1 and L2 layers and averaged over 100 iterations. While keeping the L1 weights as trained, randomization only in the L2 model neurons decreased the average modulation index to 0.05 (from 0.18; Figure 5). The difference between the L1 and L2 modulation index still remained significant (*p* < 0.000003, *t* test). This means that L1 and L2 could still be differentiated according to this metric. But note that the L2 modulation index is substantially reduced with randomization of only the L2 weights. This means that incorporating learned L2 weights leads to a much higher sensitivity to textures than random L2 weights, so learning in the second layer adds to the texture sensitivity that is developed.

We found that randomizing both L1 and L2 dramatically decreased the sensitivity of the L2 neurons to the textures versus the noise and yielded an average modulation index of −0.02 and 0.01, respectively, for L1 and L2 (Figure 5). The difference between L1 and L2 was much less significant (*p* = 0.0025, *t* test on the magnitude). In the case of randomized weights in both L1 and L2 (L1L2rand), the range of modulation indices is −0.01 to 0.03. This range is too small to capture the V2 neuron modulation indices. We see that the texture sensitivity of L2 neurons breaks in the complete absence of the trained weights and shows very low modulation similar to L1. This indicates that the sensitivity to high-order statistics like textures is not a trivial outcome of the deep network architecture. The CNN model with trained weights learned from natural images corresponds better to the neurophysiology data than the CNN architecture itself.

### Systematic quantification of the CNN to the visual brain data

**Figure 6.**
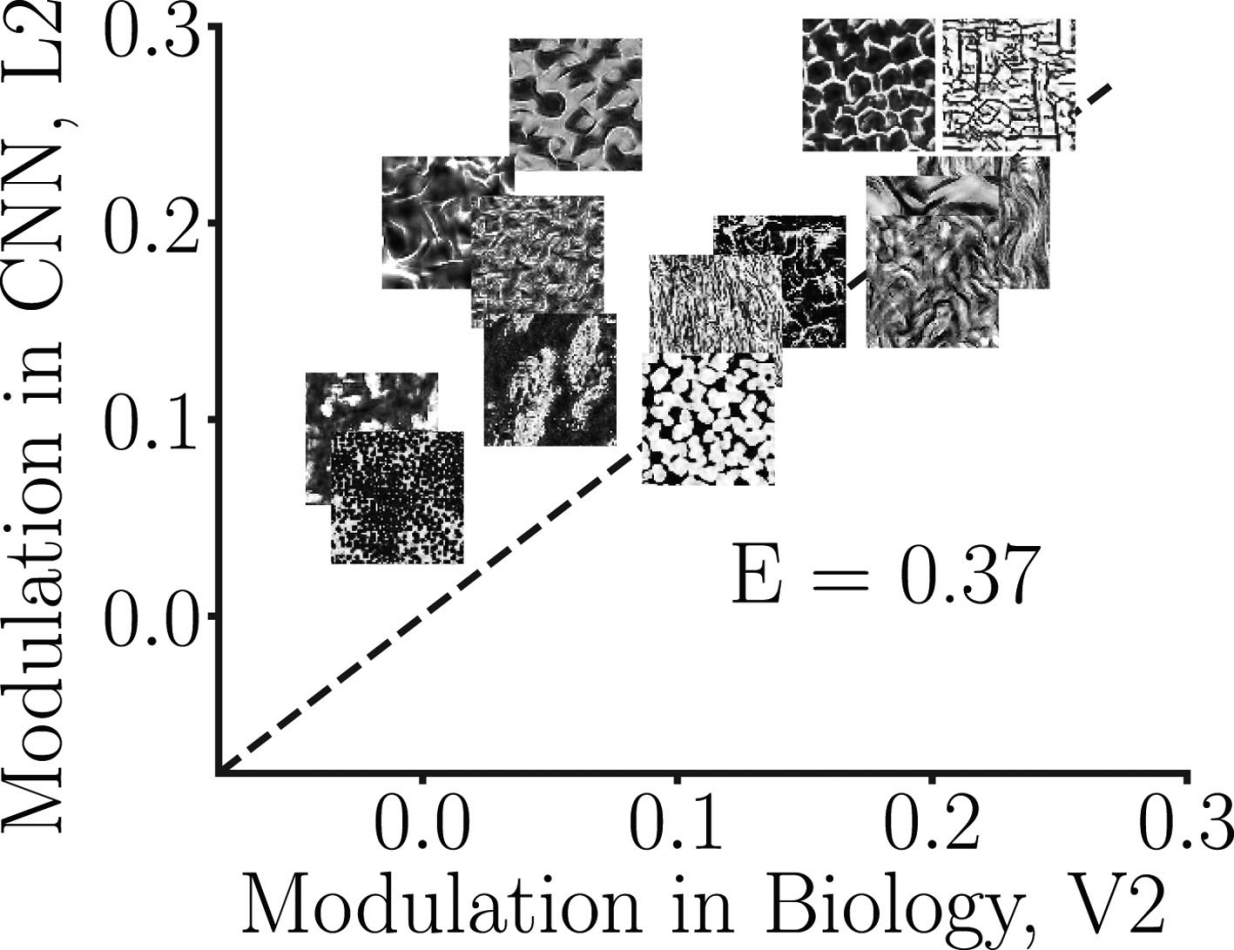
Modulation index comparison between the V2 data and a set of 103 randomly selected neurons from the CNN L2 (i.e., no fitting of the CNN to the cortical data). Random selection of model neurons does not match the neurophysiology data well (Euclidean error 0.37), prompting us to explore various subset selection methods for selecting the model neurons from the CNN population that best fit the cortical data. V2 data were collected in Freeman et al. (2013).

We have shown some qualitative correspondence between the CNN and the cortical data. Figure 6 illustrates that for a random selection of CNN model neurons, indeed this correspondence is only qualitative. The mean modulation indices for the various textures in the V2 cortical data versus the L2 in the CNN are correlated (Spearman's rank-order correlation is 0.65). We have also seen that the average correlation across different random CNN populations of 103 neurons is highest in L2 and reduces in the higher layers (L1: −0.11, L2: 0.62, L3: −0.17, L4: 0.19, and L5: −0.12).

However, all the correspondence we have shown thus far used the CNN model as is, without fitting any aspect of the CNN to the cortical data. We therefore wanted to know if one can obtain better correspondence to the data by optimizing the choice of model neurons to best fit the data. Other work has, for instance, considered linear weighted combinations of CNN model neurons to best fit cortical data (see, e.g., discussion in Kriegeskorte, 2015).

Here, we wondered whether there exists a set of 103 CNN model neurons that can fit well the cortical data. That is, rather than considering all model neurons in the CNN or some random selection of neurons, we posited that perhaps a particular subset of the neurons could better explain the subset of experimentally recorded V2 neurons. Recall that the choice of 103 model neurons is to match the neurophysiology data. The modulation index for the simulations, similar to the neurophysiology data, is computed as an unweighted average over the 103 model neurons (therefore our focus on equally weighted model neurons). We considered finding a subset of model neurons that best fit the modulation indexes from electrophysiology data, though we also compared to the optimal fit that includes all model neurons with different weights.

We view this question of obtaining a quantitative fit to the neurophysiology data for the modulation index as complementary to the more qualitative approach we used above. The qualitative approach gave us an indication of compatibility of the CNN to the neurophysiology data, on average, and highlighted some main trends, that is, that texture sensitivity emerged in the CNN in the second layer and that using weights learned from natural images in the CNN led to more sensitivity to textures than considering the CNN architecture alone and not incorporating learned weights. The quantitative metric we develop next is meant to tell us how closely the CNN can quantitatively match the neurophysiology data in the neural modulation index when selecting for the same number of model neurons. For the remainder of the article, we focus on this question with respect to the modulation index metric, capturing sensitivity to the textures versus the noise.

We note that the subset selection aspect of this problem makes it different from a standard regression and from approaches we are aware of for fitting neural data, which often take a linear combination of the model neurons (Kriegeskorte, 2015; Yamins et al., 2014). Why search for a subset of CNN neurons that can fit the cortical modulation index data? Our rationale was that finding such a subset would suggest that at the population level, there is some overlap between the CNN model neurons and the cortical neurons in their representation for the texture versus the noise stimuli, as explained below.

#### Approaches for fitting CNN model neurons to the neural data

To show a systematic quantification, we probed the CNN to select a subset of 103 model neurons that is most consistent with the cortical neurophysiology experiments (Figure 1c). To show the robustness of our results, we considered several different approaches for subset selection.

First, we employed a greedy technique, which we call *subset greedy*, to choose a subset of 103 model neurons that best match the neurophysiology data from the brain. Briefly, from the set of all possible model neurons, the greedy approach chooses the first neuron with the closest Euclidean distance to the V2 mean modulation index data; then, the second neuron is added to this subset so as to minimize the Euclidean distance and so on until a total of 103 neurons are chosen (see Technical Methods).

For comparison to our greedy fitting approach, we also applied an optimal weighted average or *full population* approach. The full population approach finds a weighted sum of the model neurons (under the constraint that the weights are nonnegative and sum to 1) that is the closest in squared Euclidean distance to the experimental data. Notice that the weighted average may include all available neurons and weight neurons differently. The greedy approach is, in contrast, an approximation that finds a subset of 103 neurons with equal weights that best matches the neural data.

Our rationale is that the full population approach shows the best fit one can obtain with model neurons from a given layer. However, it does not show an actual population representation that matches the data since the neurophysiology experiment and analysis includes exactly 103 neurons equally weighted; it only reveals a linear transform of the representation. The greedy method chooses a subset of 103 neurons and thus uses the actual CNN representation. This subset selection approach is therefore more comparable to the analysis in the cortical experiments, in which the modulation index is computed as an equally weighted average of the model neurons. However, the greedy approach is suboptimal. It chooses 103 neurons in a greedy manner. We therefore wanted to compare to an optimal approach that can weight all model neurons, to see if this results in similar trends as we manipulate aspects of the model. So we applied another model selection technique, which is a regularized version of the full population fit that selects 103 neurons, and we termed it as *subset regularized*.

Insights of having three different model neuron selection techniques is like tackling the same problem from different perspectives. Landing onto the same conclusion, even though they operate in different ways in their selection of the CNN neurons, makes our investigation more comprehensive and shows that there are model neurons in deep networks that are more V2-like according to the modulation index population metric.

#### Cross validation

In the next sections, we show results for fitting the CNN neural population to the V2 texture data with these approaches. To test the generalization capability of our method, we cross-validate all the fits, that is, training the model neuron subset selection on one set of texture and noise images, and testing on the left-out images. For the cross-validation, we extended the image data set to a total of 225 texture and noise images for each family. We learned the population (e.g., of 103 neurons) using 224 texture and noise images from each family for the training and made a prediction of the mean modulation index for the left-one-out set of 15 images. See Technical Methods section for more details about the model-fitting techniques.

#### Metrics for quantifying the CNN model fits

We quantified the CNN fits to the data with two metrics, to see if different metrics provide us with similar conclusions. First, we calculated the *Euclidean error* distance *E* between the mean modulation indices in the neurophysiological data versus the modulation indices obtained from the CNN for each family. A smaller Euclidean distance indicates a better fit to the V2 data and higher correspondence to the brain (see Technical Methods section for details). The rationale behind using the Euclidean distance as a measure of correspondence is that it is directly related to the root mean squared error (MSE) up to a normalization constant. We chose an error metric in the fitting that is sensitive to absolute values rather than relative values, since we are fitting modulation indices. Our optimal weighted and subset regularized fits are done in terms of squared Euclidean distance, which for the optimal fitting method makes the error and regularization terms work at similar scales. In the subset greedy approach, MSE and Euclidean (and even squared Euclidean) distances indicate the same outcome. Second, we quantified the fits between the V2 data and the CNN using *Spearman's rank-order correlation*, in which a larger correlation corresponds to a better fit.

#### CNN L2 population fits are well matched to the V2 data compared to L1

**Figure 7.**
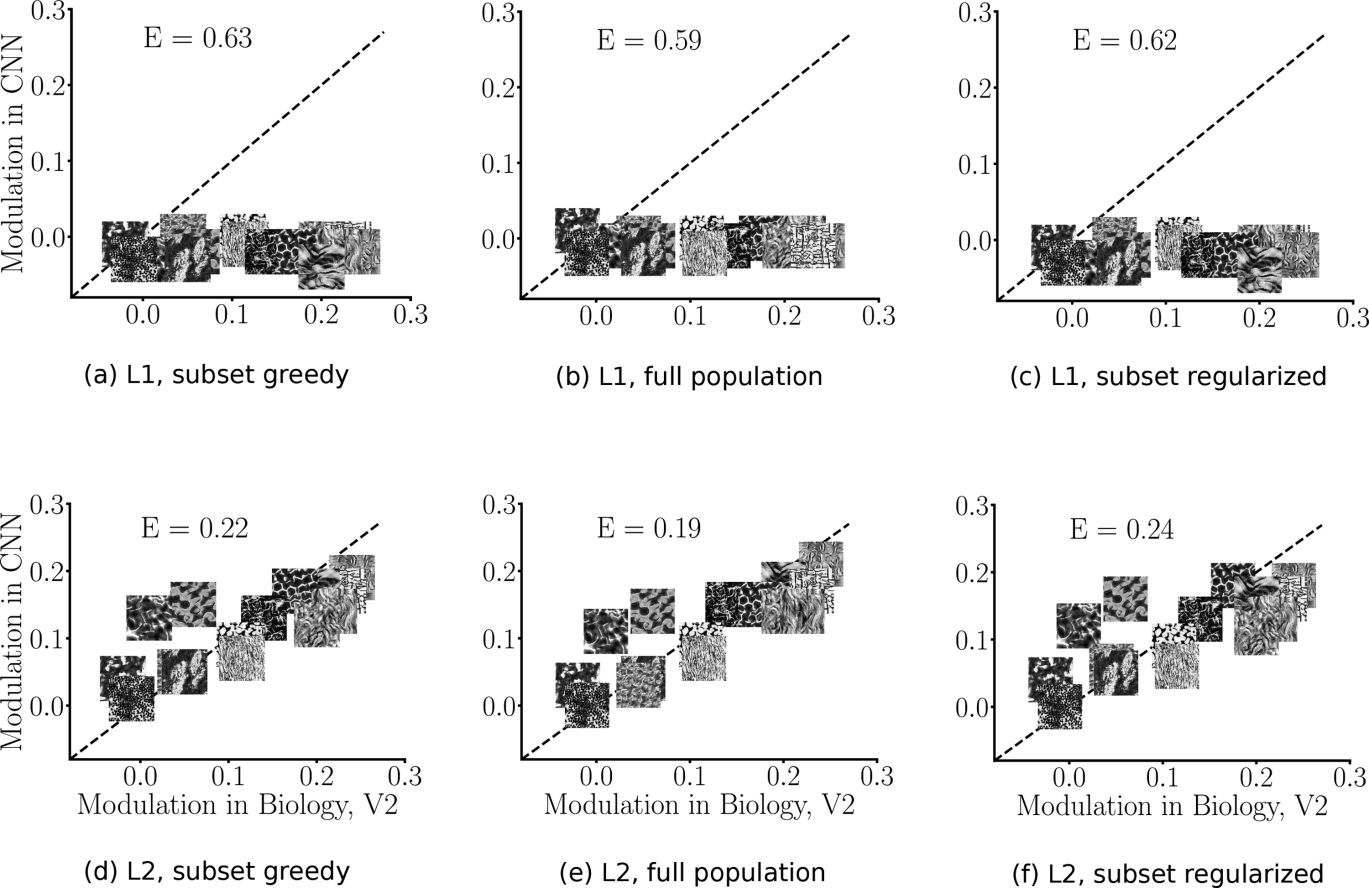
CNN model fits with cross-validation. (a-c) L1 fits with greedy subset selection (error: 0.63), full population optimally weighted (error: 0.59), and regularized subset selection (error: 0.62) fitting approaches. (d-f) L2 fits with greedy (error: 0.22), full population optimally weighted (error: 0.19), and regularized subset selection (error: 0.24) approaches. L2 neurons (second row) show superior fits to the V2 data compared to L1 neurons (first row), indicating CNN L2 neurons are more V2-like in the context of texture sensitivity. V2 data was collected in Freeman et al. (2013). Note that we plot icons of the actual textures representing each family, but the fits to the mean modulation index are based on responses to the synthesized texture images versus the noise.

We found that a subset of 103 L2 model neurons exists that provide a good fit to the V2 neurophysiology data (Figure 7, second row). Euclidean errors for the subset greedy method were 0.20 and 0.22 for the training and test predictions, respectively. Considering the whole population, we obtained train and test errors of 0.15 and 0.19, and for the regularized fits, we obtained errors of 0.20 and 0.24, respectively. In contrast, all three fitting approaches showed that for the L1 neurons, no such set exists that can fit the V2 data well (Figure 7, first row). It is interesting to note that this result held even if we did not cross-validate the data, that is, even training and testing on the same images and therefore overfitting could not lead to good correspondence of the L1 neurons with the V2 data. In addition, we could not get a good correspondence of L1 neurons with the V2 data, even when we increased the number of L1 neurons by a factor of ≈30 (103 model neurons vs. neighborhood of 8 × 8 × 48; Figure 2).

Our fitting results match the expectations based on the distributions of L1 and L2 model neuron modulation indices of Figure 4. The L1 modulation indices are all near 0, so it is not possible to find a population of L1 neurons that captures the higher average modulation indices of the V2 neurons. In contrast, the L2 model neurons have the proper range of modulation indices to capture the V2 sensitivity. Overall, our results indicated that the second layer, but not the first layer of the CNN, is better matched to the V2 data in terms of the sensitivity to textures versus spectrally matched noise.

These fitting errors were all lower than the random selection of 103 model neurons in the population that we examined earlier (compare to Figure 6; Euclidean error 0.37). The explained variance (*R*^2^) was 0.60 for the subset greedy, 0.54 for the subset regularized, and 0.70 for the full population. In contrast, the explained variance for the random population of 103 neurons was 0.40. Note that this represents a lower bound, since we are not considering the variability due to samples in a family, nor are we taking into account variability in the experiments due to stimulus repeats.

In the context of Spearman's rank-order correlation, we expect the layer to be highly correlated with V2 data if the model neurons in the layers have similar representation to the natural and noise properties in the input stimulus. Overall, for all the three methods, we found that L2 neurons show stronger correlation with the brain data than L1 neurons (0.80 vs. 0.03 in greedy, 0.86 vs. 0.04 in full population, and 0.75 vs. 0.09 in regularized selection). We see that the correlation is aligned with the Euclidean distance metric.

We have performed additional simulations, leaving a whole class of textures out and predicting them by training from the rest of the classes. This increases the fitting errors in comparison to the leave-one-out technique of cross-validation in L2 (greedy: from 0.22 to 0.32; full population: from 0.19 to 0.30; regularized: from 0.24 to 0.31). However, these errors are low in comparison to the fitting errors of L1 neurons. We have also seen a similar trend in the compatibility for the Spearman correlation (greedy: from 0.80 to 0.46; full population: from 0.86 to 0.55; regularized: from 0.75 to 0.48). The correlations therefore decrease but are still relatively high compared to the L1 neural fits. This indicates that model neurons retained by subset selection capture some properties common to all textures, not just to the textures used for the fit.

#### CNN population fits are reduced for the architecture alone, with random rather than learned weights

Given the good correspondence of the L2 model neurons to the V2 data, we wondered at what point this fit breaks or can be reduced. In the qualitative section, we found that on average, when selecting 103 model neurons randomly, the network developed more sensitivity when the weights of the CNN were learned from natural images, signifying that the learning is important beyond the architecture itself. Here we considered this question from the complementary approach of quantitatively selecting 103 model neurons that best match the neurophysiology data.

**Figure 8.**
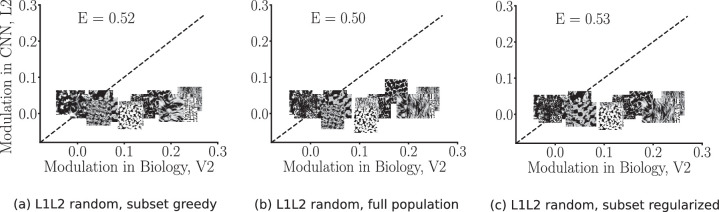
V2 fits of the CNN architecture with random weights (rather than weights learned from natural images), cross-validated. Both the L1 and L2 weights are randomly selected (hence denoted as L1L2 random). (a) Greedy subset selection fits (error: 0.52). (b) Full population optimally weighted fits (error: 0.50). (c) Regularized subset selection fits (error: 0.53). Randomization of the CNN weights of L1 and L2 leads to reduced compatibility of L2 with the V2 data (compare to Figure 7, second row).

We found that taking random weights in the CNN resulted in a larger error, and therefore a reduced fit, compared to the trained network. This can be seen in Figure 8. The Euclidean errors were 0.52, 0.50, and 0.53, respectively, for the greedy subset, full population, and regularized subset techniques.

**Figure 9.**
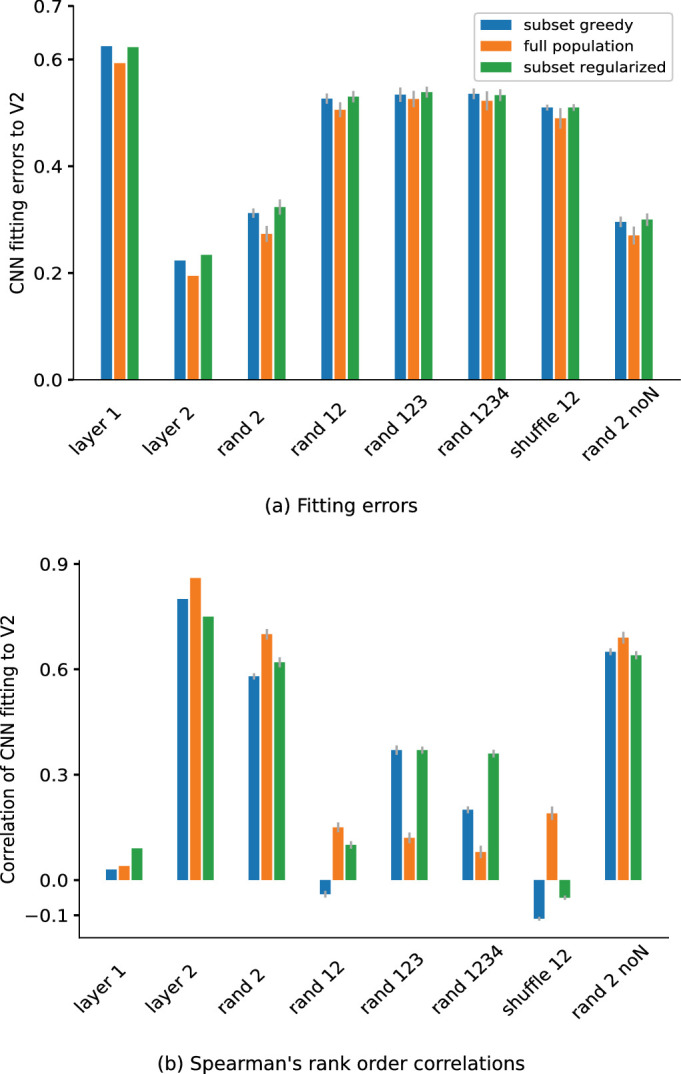
Summary of the CNN model-fitting errors to the V2 data (cross-validated), including various model manipulations. *rand 2* denotes applying random weights only for L2; *rand 12* denotes applying random weights for both L1 and L2; *rand 123* and *rand 1234* indicate that the neurons from Layers 3 and 4, respectively, are fitted. *shuffle 12* denotes shuffling the weights in L1 and L2, and *noN* denotes no normalization in the CNN layers. Errors in random and shuffled weights are averaged over 10 iterations, and error bars show their standard deviation. (a) L1 neurons (*Layer 1*) cannot fit the V2 data well (see also Figure 7, first row). For these manipulations, the errors are lowest in L2 (*Layer 2*), indicating that the second layer of the CNN could better match the cortical V2 data for the texture sensitivity (see also Figure 7, second row). Randomizing the weights in the CNN overall increases the fitting errors and hence reduces the compatibility for the texture data (see Figure 8). This indicates the importance of training the CNNs on natural scenes to develop texture sensitivity, resulting in a better match to the V2 data. (b) Spearman's rank-order correlations of the CNN model fits with the V2 data. The second layer with the trained weights shows the highest correlation with the brain data. This result is aligned with the fitting error results in (a), in which the second layer neurons show lowest fitting errors.

Figure 9 shows a more comprehensive summary of the cross-validated fitting errors (9a) and Spearman's rank-order correlations (9b) across a range of random weight controls. Errors are measured in Euclidean distance on 225-fold (leave-one-out) cross-validation. The figure first summarizes the main results on the trained weights, before showing the results for the various randomized conditions. As described in the previous subsection, the trained L1 model neurons (*Layer 1*) exhibited the highest fitting errors and lowest correlation among all layers and controls, meaning they resulted in a poor fit (hence little correspondence) to the neurophysiology V2 data (see also Figures 7a–c). The trained L2 model neurons exhibited the lowest fitting errors (greedy subset 0.22, full population 0.19, and regularized subset 0.24) and the highest correlations (greedy subset 0.80, full population 0.86, and regularized subset 0.75) in all fitting techniques, meaning that L2 achieved better correspondence to the V2 data (Figures 7d–f).

We exhaustively explored a range of controls for randomizing the CNN layer weights (Figure 9a), therefore considering the influence of the architecture alone. Overall, assigning random weights to the network layers increased the cross-validated fitting error. Randomizing only Layer 2 weights and keeping the L1 weights trained (*rand 2*) led to significantly worse fits than when both the L1 and L2 weights were trained in all three model neuron selection techniques (*p* < 0.000002 in greedy; *p* < 0.00007 in optimal; *p* < 0.00002 in regularized; one-sample *t* test). This indicated that training on the full CNN model (i.e., both the L1 and L2 weights) led to an improvement of the fit versus training on the L1 weights alone. Randomizing both Layer 1 and Layer 2 weights (*rand 12*) lead to a more dramatic increase of the errors in the fitting (see also Figure 8). This indicated that training the first layer alone went some way in obtaining a better fit. Overall, the CNN model trained on natural images had the closest fit to the neurophysiology V2 data, in line with the qualitative results that we showed in the earlier portion of the article. One can arguably say that relevant texture statistics come from interactions at particular frequencies, which cannot be captured by random weights because of their poor frequency localization.

We wondered if deeper random architectures could lead to better correspondence with the brain data. We therefore considered randomizing Layer 1, 2, and 3 weights (*rand 123*) and randomizing Layer 1, 2, 3, and 4 weights (*rand 1234*). For these conditions, we fit the outputs of Layers 3 and 4, respectively, to the data. The goal here was to see if stacking more random layers helped in obtaining a better fit to the data. However, the error remained high even when we stacked together four layers (compare *rand 12* with *rand 123* and *rand 1234*). Therefore, a deeper random network did not rescue the fit.

As an alternative to randomizing the weights, we also asked what happens if the trained weights within each filter are shuffled to destroy any spatial correlations. This maintains the distribution of the trained weights in each of the model neurons, which the network might benefit from. To test this, we spatially shuffled the trained weights for each of the model neurons in both layers (*shuffle 12*). We found that this resulted in a slightly better fit than the randomized counterpart, but still remained poor (compare *rand 12* vs. *shuffle 12*).

Overall, our results reveal the necessity of training the deep learning models on the natural image data set beforehand to achieve a better match to the V2 texture sensitivity data. Other studies have also indicated the necessity of model training (Cichy et al., 2016; although see also Jarrett, Kavukcuoglu, Ranzato, & LeCun, 2009; Saxe et al., 2011). We found that one trained layer did go some way in obtaining correspondence, but both layers trained performed the best (echoing our average results in the Differentiating L2 neurons from L1 in CNNs subsection).

#### CNN fits improve in L2 after rectification and improve further with pooling, but not local normalization

An important question regarding the CNN is how the various computations influence the compatibility of the model to the data.

One way to tease apart the different computations (conv, rectification as in the ReLU operation, pool, norm) involved in L2 is to consider the intermediate outputs of the CNN trained on the ImageNet database as in Figure 1b, and to quantify the impact of each of these on the compatibility with the V2 data. This gives us a sense of how much each of the computations contributes to capturing the texture sensitivity. We therefore generated outputs from each of the intermediate points in L2.

Outputs from *conv2* (i.e., after only the convolution in the second layer) had high fitting errors. This is because the response from the conv layers can be negative before the ReLU. We found that compatibility to the V2 data starts to develop already after the rectification in the second layer (i.e., ReLU2). This is indicated by the Euclidean fitting error (greedy: from 0.62 in L1 to 0.33 in L2; full population: from 0.59 in L1 to 0.31 in L2; subset regularized: from 0.62 in L1 to 0.34 in L2). The fitting errors after *pooling* improved (subset greedy 0.22; full population 0.20; subset regularized 0.24). After the local normalization (i.e., the point in L2 that we initially referred to in all our measurements), the fitting errors were subset greedy 0.22, full population 0.19, and subset regularized 0.24. The main improvement in the fit appeared to be at the L2 rectification (ReLU) stage.

We also see the same trend in the Spearman correlations. The main improvement in the correlation happens after the ReLU2 stage (greedy: from 0.03 in L1 to 0.48 in L2; full population: from 0.04 in L1 to 0.48 in L2; subset regularized: from 0.09 in L1 to 0.55 in L2). Compatibility keeps increasing in the pooling stage in L2 (greedy subset 0.75, full population 0.82, and regularized subset 0.74). If we go to the next stage and take the output from the normalization operation, the correlations remain almost the same but with a slight improvement in all the fitting techniques (greedy subset 0.80, full population 0.86, and regularized subset 0.75). Overall, we obtained the best fit to the neurophysiology data by incorporating the L1 computations, followed by the L2 convolution, rectification, and pooling.

In the local response normalization in L1 and L2, each neuron response is divisively normalized by the responses of five neurons (including the self) that spatially overlap. This loosely mimics the divisive suppression in cortical V1 neurons (Albrecht & Geisler, 1991; Geisler & Albrecht, 1992; Heeger, 1992). Since normalization is widely used in V1 models, we wanted to further investigate its impact on the result for the textures: For instance what happens to the sensitivity if we ignore the local normalization of L1 (*norm1*) and L2 (*norm2*) layers altogether?

As one way to gauge what happens when we remove normalization from the network, we retrained AlexNet on the ImageNet database but with the local normalization layers removed. From an object recognition perspective, removing the normalization layers in the CNN model decreased the accuracy with a small margin (from 57.0% to 55.71%), echoing previous observations. Fitting errors with and without normalization were subset greedy: 0.22 versus 0.30; full population: 0.19 versus 0.27; and subset regularized: 0.24 versus 0.30. This indicated that the trained model with local normalization resulted in a better fit than the model trained without normalization.

We also examined removing normalization from the CNN with random L2 weights, following the approach of the previous subsection. We found that the local normalization had a mild impact on the compatibility with the neuronal data. This can be seen in Figure 9a (*rand 2* vs. *rand 2 noN*; *p* < 0.001 in greedy, *p* = 0.70 in full population, which was high and did not pass significance, and *p* < 0.009 in subset regularized; independent sample *t* test). Taken together, our results suggest that normalization had only a mild role in improving compatibility for the texture data.

#### Higher layers of the CNN further, though modestly, improve the V2 data fits

So far, our main focus has been when sensitivity to textures first develops, strongly differentiating L1 from L2. However, it is interesting to consider how this changes across higher levels of the deep network.

On one hand, an improvement in higher layers might be expected, since the CNN learns progressively, and computations in visual cortex may be better approximated by multiple layers. There need not be a one-to-one fit between the CNN layers and the cortical areas. On the other hand, an increase in error might be expected at high layers, similar to the observation that area V4 better discriminates synthesized “jumbled image” textures than area IT (Okazawa et al., 2015; Rust & DiCarlo, 2010).

The modulation index values for L3, L4, and L5 are −0.12, 0.05, and 0.01, respectively. Even though higher layers show lower mean modulation index, there is enough diversity in the distribution of modulation indices for these layers to produce a good fit with the subset selection approaches.

**Figure 10.**
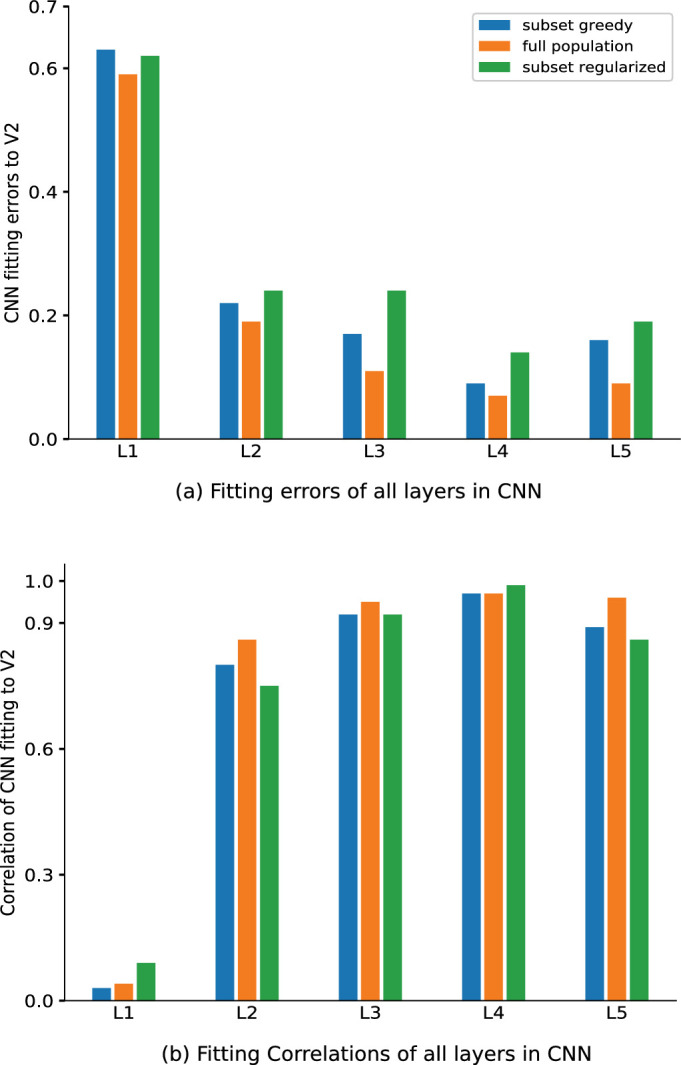
Fitting errors and Spearman's rank-order correlations for all the layers in AlexNet. Both the decrease in error (a) and increase in correlation (b) are most prominent from L1 to L2, with modest improvement thereafter.

The results for both Euclidean errors (greedy: 0.17, 0.09, 0.16; full population: 0.11, 0.07, 0.09; regularized: 0.24, 0.14, 0.19) and Spearman's rank-order correlations (greedy: 0.92, 0.97, 0.89; full population: 0.95, 0.97, 0.96; regularized: 0.92, 0.99, 0.86) are summarized in Figure 10. We found that for higher layers of the CNN beyond L2, the fit modestly improved, as seen by both the Euclidean error and Spearman's correlation. However, it then decreased again in the highest layer.

#### The VGG network, with smaller RF sizes, develops compatibility to the V2 data more gradually

We have thus far focused on the AlexNet CNN, and showed that L2 (but not L1) model neurons develop texture sensitivity similar to what was found for the V2 data. Our main approach was to take a single popular base model and its computational building blocks, and examine how manipulating certain pieces impacts the compatibility of the CNN model with the V2 neurophysiology data. We also applied our methodology to the VGG16 model (Simonyan & Zisserman, 2015) (which we denote VGG), trained on the same ImageNet data set. In the VGG model, the receptive field sizes are smaller than in AlexNet and develop more gradually; we wondered if that would influence when texture sensitivity emerges in the network.

We found that the VGG develops texture sensitivity more gradually than AlexNet. Starting from L1, the fit kept improving, with the largest reduction in the error between L3 and L2 (similar to the L2 to L1 comparison in AlexNet). The Euclidean errors quantify this trend (Table 1). In particular, between L2 and L3, the errors reduced from 0.42 to 0.15 in greedy, from 0.33 to 0.12 with the full population, and from 0.43 to 0.17 in the subset regularized technique. The fit for L4 remained similar to L3, with some increase in error for L5 with the subset selection methods.

**Table 1.** Euclidean error distances between the model fits and V2 neurophysiology data, in all layers of VGG net (cross-validated). The most significant reduction in fitting errors (hence increase in correspondence with V2) happens in L3 when the RF size becomes more compatible with V2.

	VGG net				
Approach	L1	L2	L3	L4	L5
Subset greedy	0.63	0.42	**0.15**	0.10	0.21
Full population	0.61	0.33	**0.12**	0.06	0.03
Subset regularized	0.62	0.43	**0.17**	0.20	0.35

Since the VGG neurons have a smaller receptive field size than AlexNet, we wondered if further downsampling the textures to obtain a more realistic RF size match between L2 and V2 would improve the result. However, downsampling further to make the L2 receptive field size more compatible with V2 showed unreasonably high fitting errors, perhaps because in this case, the images become too blurry and partially lose their texture properties.

This means that L3 in VGG becomes more comparable with L2 in AlexNet (and to V2 in the brain). This is probably because the increase of the RF size in AlexNet is more rapid than in the VGG. L2 RF size in AlexNet is 39 × 39 (with stride = 2 in L1), but the same in the VGG net is only 16 × 16; in contrast, the RF size in L3 of the VGG net is 44 × 44. This is also consistent with the observation that the modulation index for the texture images computed over the Portilla and Simoncelli (2000) parameters is weaker and more variable at small sizes (Ziemba, Freeman, Simoncelli, & Movshon, 2018; Ziemba, Perez, Simoncelli, & Movshon, 2017). The VGG also does not include normalization, but based on our manipulations with AlexNet, we believe that its effect is small for the trained network in terms of compatibility with the V2 data.

In sum, for the VGG network, texture sensitivity developed more gradually; compatibility with the texture data first emerged in L3, and the change between L2 and L3 was the most striking. Overall, we found that middle layers in the VGG showed better compatibility with the biology.

## Summary of results

We focused on the neurophysiology data in Freeman et al. (2013) & Ziemba et al. (2016), which showed that texture sensitivity develops at the level of V2 but not V1. To compare the CNN model and cortical data in terms of texture sensitivity, we initially examined some qualitative approaches (without any fitting), including visualization of the texture class clustering (Figure 2) and examination of the mean modulation index (Figure 3). This then prompted our quantitative approach for fitting the cortical data, by determining model neurons that best fit the data.

Our main goal was to quantify the compatibility between the CNN and the cortical texture data, and to understand what factors and computations impact this compatibility. We wanted to both pinpoint when (in terms of layer and computation) the compatibility first emerges, how it changes across layers, and what other factors (e.g., training, receptive field sizes) are important. Our main findings were as follows:

• The CNN L1 was not compatible with the V2 texture data (Figures 7a–c), as revealed by the large Euclidean error and small Spearman's rank-order correlation in the model fits (see Figures 9a and 9b).
• The CNN L2 showed a marked decrease in error and increase in the Spearman's correlation in fitting the V2 data (see Figures 9a and 9b).
• The compatibility between L2 and V2 was first observed following rectification and improved with pooling; local normalization only had a mild effect.
• The compatibility between L2 and V2 showed some modest improvement for higher layers of the CNN and then slightly decreased again in L5, as quantified by the Euclidean error and Spearman's correlation (see Figures 10a and 10b).
• The CNN training on natural images was important to obtain compatibility between L2 and V2; randomizing or shuffling the weights reduced this compatibility (see Figures 9a and 9b).

## Discussion

We chose the CNN class of model for our comparison to brain neurophysiology data because it stacks some of the same basic building blocks as other cortical models, because previous visualizations have shown that it learns rather rich structure in the second layer, and because recent work has shown intriguing compatibility with cortical data along the hierarchy. Our goal was not just to find compatibility with V2 data but rather to use the CNN model as a tool with which to explore these computational building blocks and other aspects of the model.

Here, we specifically focused on the transformation between V1 and V2. Since this is early along the cortical hierarchy, we were particularly interested in getting a handle on the layers and respective computations (e.g., convolution, rectification, spatial pooling, normalization) for which the CNN model can develop texture sensitivity and obtain better compatibility with the V2 neurophysiology data.

In this sense, we are thinking of the CNN as going beyond a black box. Rather, we would like to know when compatibility first emerges in the CNN, how it progresses along the layers, and what are the important computations and factors (e.g., having the model trained, keeping the RF size similar) that impact our results. We specifically focused on texture processing in early areas of visual cortex, for which Freeman et al. (2013) have compellingly shown develops in V2 but not in V1. This provided a rich data set to quantitatively compare the CNN and other related classes of hierarchical models.

We presented initial versions of this work and the ability of deep neural networks to qualitatively capture some of the V2 versus V1 texture data in abstract form (Laskar, Sanchez Giraldo, & Schwartz, 2017). Ziemba et al. have shown that a descriptive model of V2 can capture some of the qualitative results on the increased sensitivity to naturalistic textures (Ziemba, Goris, Movshon, & Simoncelli, 2015; Ziemba, 2016). Zhuang et al. showed increased sensitivity to textures versus noise in higher layers of deep neural networks, and related this to sparsity (Zhuang, Wang, Yamins, & Hu, 2017). The work described here, in contrast, focuses on quantitatively fitting the experimental data to changes that develop across the first two cortical areas.

We first asked at what layer and for what computations texture sensitivity first emerges in the AlexNet. We found that L2 (but not L1) of AlexNet could well fit the V2 data. More specifically, we found that texture sensitivity first emerged in the second layer of AlexNet following the rectification (i.e., ReLU2; Figure 1b) stage of L2 and that this was improved by pooling. Local response normalization did not significantly impact the emergence of the texture sensitivity in the model.

What factors are important for better compatibility between the CNN and the cortical V2 data already in the second layer? We found that training on natural images was necessary for the model to develop compatibility with the cortical data. Various manipulations of random or shuffled weights could partly account for the modulation index data but lead to a reduced fit between the CNN model and the V2 data relative to the learned weights. This indicated that the architecture by itself was not sufficient to obtain good compatibility.

Incorporating a trained first layer but random weights in the second layer still did not yield as much texture sensitivity or compatibility with the V2 data as when both layers were trained (see Figures 4 and 8). However, when the first layer was trained, the compatibility was much better than when both layers were random. The importance of retaining the trained weights (rather than random weights) in the first layer may be because the model neurons need to be matched to the frequencies and orientations that appear more often in the natural images in order to pick out the higher-order structure of the textures in the subsequent layer.

In addition, we found that the receptive field size needed to develop sufficiently fast (comparing AlexNet and the VGG) and that the local normalization in AlexNet only had a limited role in obtaining good fits to the texture data.

Although the AlexNet CNN showed compatibility with the V2 data, it too had some deviations from the cortical data. For instance, according to the qualitative results in Figure 2, it is intriguing that, with the same amount of model neurons, the brain V2 outperforms the CNN L2 at grouping together different texture families (for the brain data, refer to Figure 4 in Ziemba et al., 2016). In addition, as indicated by the modulation index, the CNN on average was more sensitive to the textures versus the noise than the V2 population. Its rank order of the sensitivity to the textures on average also deviated from the data. This suggests that the CNN still has room for improvement in terms of capturing the cortical data.

Local response normalization is a computation prevalent in visual cortex (Carandini & Heeger, 2012). It is possible that the limited role of normalization in obtaining compatibility with the V2 data is due to the homogeneous nature of the textures. Divisive normalization may play a more important role in capturing data for nonhomogeneous images. Therefore, future work should test compatibility with V2 data over a broader range of natural stimuli and tasks.

In addition, the local normalization model we implemented is based on AlexNet and limited to an equally weighted normalization of groups of five filters. Divisive normalization models of cortex have been studied extensively (Albrecht & Geisler, 1991; Geisler & Albrecht, 1992; Heeger, 1992; Carandini & Heeger, 2012). Future studies should incorporate richer biologically inspired models into hierarchical architectures and consider their compatibility with cortical data. For example, in cortical studies, normalization is strongest for the same orientation as the RF (see Geisler & Albrecht, 1992, for measuring normalization with stimuli at the optimal orientation; Günthner et al., 2019, for recent CNN model fitting to V1). Weighted divisive normalization has been incorporated in models of image statistics (see, e.g., Schwartz & Simoncelli, 2001), including multilayer models (Ballé, Laparra, & Simoncelli, 2015). One possibility for improvement of the V2 modeling in future work is incorporating into CNNs rich models of surround normalization (i.e., going beyond a local normalization within the classical receptive field; see, e.g., the range of surround normalization models used for modeling V1 data in Coen-Cagli, Kohn, & Schwartz, 2015). Models of surround normalization have been recently incorporated in CNNs (see e.g., Ren, Liao, Urtasun, Sinz, & Zemel, 2016; Sánchez Giraldo & Schwartz, 2019).

More broadly, we have compared supervised learning to no learning at all. Based on our results, we do not expect that a single layer of unsupervised sparse coding followed by random weights into the second layer would sufficiently account for the V2 texture data. This also in and of itself does not lead to rich second-layer receptive field structure. However, two-layer unsupervised hierarchical models have led to more interesting receptive field structure in the second layer and have shown some compatibility to V2 data (e.g., Lee, Ekanadham, & Ng, 2008; Coen-Cagli & Schwartz, 2013; Hosoya & Hyvarinen, 2015). There is indeed much room and interest to examine such more sophisticated unsupervised learning models in the future and their compatibility with the texture data. A thorough comparison between supervised and unsupervised learning models is an important future direction.

In addition, adding more neurons per layer may also play a role, and we found that choosing from a larger spatial neighborhood could improve the CNN fit. However, larger spatial neighborhoods did not reveal a good fit for the L1 neurons in the AlexNet, and the first layer of all models was not compatible with the V2 data. This also resonates with the original texture model of (Portilla & Simoncelli, 2000) that was actually used to generate the experimental stimuli; although the model does not have an explicit V2 neuron representation, the textures are generated by joint statistics between V1 model neurons, that is, by a two-layer model. Though we have not exhausted all the possible hierarchical models, our method is pragmatic enough to be applied to any hierarchical models to test and find correspondence with the neurophysiology data.

While we have focused on texture sensitivity in V2, there is room to explore compatibility of CNNs with changes across the early cortical hierarchy for other stimulus properties. For instance, neurophysiology studies have found selectivity in V2 to conjunctions of orientations and to figure ground (Ito & Komatsu, 2004; El-Shamayleh & Movshon, 2011; Zhou, Friedman, & Von Der Heydt, 2000), with some aspects addressed in computational models of V2 (Lee et al., 2008; Coen-Cagli & Schwartz, 2013; Hosoya & Hyvarinen, 2015; Heeger, Simoncelli, & Movshon, 1996; Zhaoping, 2005; Hegde & Essen, 2000). There may not be one unique CNN architecture that explains the neural data, but we believe that testing across fairly early visual areas (e.g., V1 and V2) with less stacked computations, and for a wider range of stimuli and tasks, can facilitate understanding of the critical factors (e.g., training) and computations (e.g., convolution, pooling, rectification). Beyond area V2, studies have examined the compatibility of CNN model neurons across the different layers with shape tuning properties in visual area V4 (Pospisil et al., 2016).

In the quantitative comparisons between the modeling and data, we developed approaches for subset selection. These were more appropriate for the given cortical data than a linear combination of the neural population responses, which is typically used in fitting data. This is because the subset approach more faithfully represented the data analysis, which included an equally weighted average modulation index. This approach also allowed us to ask the question about whether there exists a population of model neurons in the CNN that can well represent the experimental data. We therefore chose a neural population from the representation itself rather than a linear transform of the representation. By finding a subset of model neurons that are most compatible with the data, it may be possible in the future to drive new experiments in which stimuli are generated from this population of model neurons and tested on the data. This may be applied more generally in the future to modeling other data sets and neural areas.

On one hand, our results add to the intriguing findings that CNNs trained on natural images have some compatibility with neurophysiology data, and moreover, we found that this holds across low levels of the cortical hierarchy. But we believe that our approach goes beyond showing compatibility, by providing a direction for manipulating these early layers and teasing apart what aspects of CNNs, the training, and computational building blocks are most critical. Our approach can also be applied to other related architectures, computational building blocks, stimuli, and neural areas (code is available in GitHub). This creates the opportunity for more discussion and systematic study of the various building blocks of deep networks and opens the door to answer the long-standing research question about correspondence between primate and machine vision.

## Technical methods

### Texture generation

For the CNN simulations, we used the same ensemble of synthetic texture images as in Freeman et al. (2013) and Ziemba et al. (2016). The synthetic images were grayscale images of size 320 × 320 and generated from an original set of 15 texture images. From each original texture, multiple synthetic texture images that matched the statistics of the original image were generated. Naturalistic textures for a given family were generated each with a different random seed, using an iterative process of constraining Gaussian white noise images to have similar marginal and joint statistical properties of the original textures (Portilla & Simoncelli, 2000). Spectrally matched noise images were generated by randomizing the phase, that is, computing the Fourier transform and inverse Fourier transform after phase randomization. From the 15 original textures, we have 15 different samples from each family, resulting in a total of 225 images of naturalistic textures and 225 images of spectrally matched noise, as used in Freeman et al. (2013) & Ziemba et al. (2016). For cross-validation, we generated extra images per texture family from the model of Portilla & Simoncelli (2000)).

### Matching receptive fields with the physiology data

We wanted to match as much as possible the spatial extent of the images that the model receptive field (versus the typical experimental neuron) is sensitive to. The input images were size 256 × 256, and the average receptive field size for V2 was approximately 150 × 150 (with the V1 receptive field approximately half that size). The receptive field size of model neurons in AlexNet is 39 × 39 for L2 and 15 for L1. We downsampled the input images by a factor of 4 to obtain images of size 64 × 64, so that the effective size of the L2 receptive field was closer to the neurons recorded from in the experiment. We could not get an exact match, due to the constraint of downsampling by factors of 2. We also originally ran the whole set of simulations without downsampling the images at all, and the results remained qualitatively similar, except that there were light improvements in the compatibility to the data with the appropriate downsampling.

Following the experiments, we contrast normalized the images before feeding them to the networks. The luminance (*l*) is given by the mean pixel intensity of the downsampled image (*I*_*d*_). The contrast (*c*) is given by the standard deviation. The contrast normalized images (*I*_*n*_) are then defined as follows:

(3) $$I_{n}=\alpha \frac{I_{d}-l}{c}+\beta ,\;$$

where the desired contrast α defines the range of the input pixels and the desired luminance β defines the intensity centered on the range. We use 0.22 as the value of α and since the desired luminance is gray, we use 0.5 as the value for β.

### Deep CNN models for texture simulations

In our simulations, we mostly used the pretrained AlexNet model, trained on natural images and specifically on the ILSVRC 2012 ImageNet (Russakovsky et al., 2015) data set. We also retrained the network on ImageNet, or on the Places365 database (Zhou, Lapedriza, Khosla, Oliva, & Torralba, 2017), yielding similar results. We also contrasted this with an equivalent model architecture that included random weights (in the interval [−1, 1]) rather than pretrained weights. AlexNet consists of five convolution followed by three fully connected layers. The first and second convolution layers are followed by local response normalizations and max-pooling. We used CaffeNet, which is a variant of AlexNet, where normalization follows the pooling. We refer to this as AlexNet for convenience. We examined the outputs from the first and second normalization layers (along with a more exhaustive examination of other layers) and compared them to the experimental data for V1 and V2 neuron outputs.

We used a modified version of AlexNet by changing the stride at L1 from 4 to 2. This allowed us to significantly reduce the receptive field size in L2 (from 67 × 67 to 39 × 39; with L1 of size 15 × 15), making it more comparable with the neurophysiological ratio of V2 to V1 RF size. This modification also matched the experimental data better in our simulations.

In addition, we simulated the response of the first 48 (instead of 96) L1 model filters as they are the ones that show orientation selectivity; the remaining are more color selective. These 48 filters are the input to the first 128 (out of 256) filters in Layer 2, so we considered these first 128 filters from L2. We focused mostly on the center four (2 × 2) spatial positions from each of these selected CNN filter responses. This was to include filters that cover the center of the input stimuli. We also tested our method on larger spatial neighborhoods and obtained qualitatively similar results.

We later considered the VGG16 network (Simonyan & Zisserman, 2015), trained on the same ImageNet data set as AlexNet. VGG16 is a 16-layer network stacked with multiple (usually 2 or 3) convolution-rectification layers with 3 × 3 filters and then followed by a pooling layer. We examined outputs from those five pooling layers, which we refer to as L1 through L5.

### CNN population fitting and subset selection approaches

We considered the total number of neurons in the CNN as the number of filters in a given layer (e.g., 48 for Layer 1 and 128 for Layer 2), times a center 2-by-2 spatial neighborhood. The rationale was that experimental data can be collected for receptive fields at different spatial positions. We chose a 2-by-2 spatial neighborhood, and did not find a significant difference when exploring larger spatial neighborhoods. We selected 103 model neurons from L2 and 102 from L1 to match the population numbers in the neurophysiology experiments of Freeman et al. (2013). Before starting the subset selection procedure, we removed from consideration the CNN model neurons that had zero response to any family. This amounted to 432 out of a total of 512 neurons from which we selected the subset of size 103.

#### Subset greedy approach

We consider the subset greedy technique known as *forward selection* to choose a subset of 103 model neurons that best match the data from the cortical neurons. In the greedy approach, the goal is to build a subset incrementally by adding neurons, one at the time, that in conjunction with previously selected neurons minimizes the Euclidean distance between the neural data and the CNN model modulation indices. This incremental process continues until we have chosen 103 neurons from the available CNN layer population. The approach is greedy, because it optimizes the selection of the next neuron as best it can given the current set of neurons. However, it does not guarantee a globally optimal solution.

Formally, let t be a 15-dimensional vector containing the average modulation indices per texture family from the 103 recorded neurons in the physiological experiments, A be the set of *n* CNN model neurons, and $m_{j}$ the average modulation indices per texture family of the *j*th simulated neuron in A. Starting from $S^{\left(0\right)}=\emptyset$, the greedy algorithm adds a neuron to the current set of selected model neurons that minimizes the squared Euclidean distance between the neuronal data and model average modulation indices:

$$S^{\left(k\right)}=S^{\left(k-1\right)}⋃\underset{j\in A\setminus S^{\left(k-1\right)}}{\mathrm{argmin}}{∥t-\frac{1}{k}\sum_{j^{'}\in S^{\left(k-1\right)}⋃j}m_{j^{'}}∥}_{2}^{2}.$$

The above procedure is repeated until the desired number of model neurons is obtained. In particular, we stop at 103 neurons. Fitting of this subset selection technique is shown in Figure 7 (first column).

#### Optimal weighted average or full population

In this approach, we find a weighted average of the set of simulated model neurons that best fit the neurophysiology data by solving a constrained optimization problem. The constraint guarantees that the sum of the weights add to 1. This approach does obtain an optimal solution and therefore shows the best one can do. However, note that it does not as faithfully capture the analysis of the neural data, since for the data analysis, an equally weighted average of 103 neural responses give rise to the modulation index data.

Formally, we have

(4) $$\begin{array}{cc} \underset{w}{\mathrm{minimize}} & {∥Mw-t∥}_{2}^{2}\\ \mathrm{subject}\;\mathrm{to} & w_{i}\geq 0,\;i=1,...,n\;\;\;\;\mathrm{and}\\ & \sum_{i=1}^{n}w_{i}=1, \end{array}\;$$

where $M=\left[m_{1},m_{2},\cdots ,m_{n}\right]$ is the matrix of average modulation index values for all families computed according to (2), and w is the vector of weights for all the *n* simulated model neurons. In terms of Euclidean distance, this is the best fit that could be attained by considering a weighted average on the simulated neurons. Nevertheless, note that the solution need not be sparse since there is no mechanism forcing the weights to become zero, and the disparity of the weight values can be hard to interpret. Fitting results of this technique is shown in Figure 7 (second column).

#### Subset-regularized average followed by threshold

As noted above, for the optimal weighted average, weights *w*_*i*_ can be very disparate. Since we seek to select a subset of the simulated neurons whose regular average (all weights are equal) follows closely the physiological experiments, we relax the selection problem by solving a regularized version of the optimization problem (5) as follows:

(5) $$\begin{array}{cc} \underset{w}{\mathrm{minimize}} & {∥Mw-t∥}_{2}^{2}+\lambda \;{∥w∥}_{2}^{2}\\ \mathrm{subject}\;\mathrm{to} & w_{i}\geq 0,\;i=1,...,n\;\;\;\;\mathrm{and}\\ & \sum_{i=1}^{n}w_{i}=1, \end{array}\;$$

where λ > 0 is the trade-off parameter that promotes weight equalization. For λ = 0, which is equivalent to solving (4), we found that only 14% of the simulated model neurons have the weights *w*_*i*_ ≥ 2*e*^−3^ with only a handful of them containing large values that account for $\sum_{i}^{n}w_{i}=1$. As λ increases, the regularization term pushes the weights towards the center of the simplex. For instance, for λ = 0.8, we found that approximately 40% of the model neurons have weights *w*_*i*_ ≥ 2*e*^−3^. The subset of model neurons is selected by applying a threshold to the estimated weights, as proposed in Li, Sundar Rangapuram, and Slawski (2016), and then choosing the 103 neurons with the highest weights. However, a main difference from Li et al. (2016) is that our two-stage procedure is applied to the solution of (5) instead of (4). This approach also yields an excellent fit to the V2 data for L2 model neurons, as shown in Figure 7 (third column). We used λ = 0.8 for all fits; lower λ increased the fitting error but did not alter the trends (and vice versa).

### Cross-validation

For the cross-validation, we extended the image data set. In the original data, there were only 15 images generated in each family. We therefore extended the set by generating 210 additional images (texture and noise) from each of the texture families. We optimized the learning, assuming that each group of 15 images, out of the 225 in each family, should yield an average modulation index that is as close as possible to the mean modulation index for that family in V2. Therefore, for 225 images in each family, we randomly divided the images into groups of 15. This yielded 15 data points per family and a total of 225 data points for all 15 families. We then applied a 225-fold leave-one-out technique, training on 224 points and leaving out one point (corresponding to leaving out one set of 15 images). We thus learned the population (e.g., of 103 neurons in the greedy subset selection method) with the 224 training data points and made a prediction of the mean modulation index for the left-one-out set of 15 images.

### Euclidean distance as the measurement of correspondence

To quantify the error between the CNN model and the neurophysiology data, we use the Euclidean distance metric. We calculate the Euclidean distance between the values computed from the CNNs and the neurophysiology V2 data. For the CNNs, the computed values include the modulation indices obtained from the various fitting and subset selection techniques, or predicted modulation index in the case of the cross-validation.

Given two vectors $x=\{x_{1},\;x_{2},\;\cdots ,\;x_{n}\}$ and $y=\{y_{1},\;y_{2},\;\cdots ,\;y_{n}\}$ are two points in Euclidean *n*-space, the Euclidean distance d(x,y) is computed using the 2-norm as follows:

(6) $$d\left(x,y\right)=\sqrt{\sum_{i=1}^{n}{\left(y_{i}-x_{i}\right)}^{2}}.\;$$

In all our experiments, *n* is usually 15, the number of texture families, as we take the average over samples and/or model neurons. Lower Euclidean distances indicate a better fit of the model to the V2 data and therefore higher correspondence of the model to the brain.
